# Sexuality, gender identity, and relationship conflict in women with ADHD in women

**DOI:** 10.1192/j.eurpsy.2026.10287

**Published:** 2026-07-31

**Authors:** D. Wynchank

**Affiliations:** 1 ADHD in Adults, PsyQ, The Hague; 2 Psychiatry, Amsterdam UMC, Amsterdam, Netherlands

## Abstract

**Introduction:**

Sexual health in neurodevelopmental disorders remains understudied. Women and transgender/gender diverse (TGD) adults with ADHD may face elevated risks of sexual dysfunction and interpersonal violence, alongside gender-specific barriers to care. Evidence also suggests higher ADHD symptom burden in TGD populations, yet little is known about sexuality and relationship safety in these groups.

**Objectives:**

Using preliminary findings from the International Survey on Experiences, Health, and Barriers in Women with ADHD, the objectives are to:

1. Describe gender identity, sexual orientation, and relationship characteristics in women/TGD respondents with ADHD.
2. Estimate self-reported sexual problems and interpersonal conflict (including intimate partner violence, IPV), and explore associations with ADHD context.

**Methods:**

Cross-sectional, descriptive analysis of an international online survey (Dutch/English) targeting women ≥18 years with self-reported ADHD symptoms or a formal ADHD diagnosis. Recruitment: social media, patient organisations, newsletters, research networks. Outcomes included demographics, sexual/romantic orientation (multi-select), relationship status, partner ADHD, and conflict tactics/IPV items. Analyses summarised distributions using valid-percent denominators, with explicit reporting of missing data due to optional items.

**Results:**

Nearly 6,000 respondents completed the survey (N=5,893). Most were assigned female at birth (n=4,114). Respondents reported diverse sexual and romantic orientations, with around two-thirds identifying as straight and over one-third as bisexual, pansexual, queer, asexual, or other minority orientations. Two in five were married, and a notable proportion reported partners with ADHD.

Although only 1% reported a diagnosed sexual disorder, this was likely underestimated due to nonresponse. Reports of relationship conflict were concerning: 15% disclosed partners insisting on sex without force, and 2.6% reported forced sex at some point. Very few endorsed perpetration themselves.

**Conclusions:**

These preliminary findings highlight substantial diversity in sexual orientation, frequent partnerships where ADHD is present, and notable rates of sexual coercion and forced sex among women/TGD adults with ADHD. The low rate of diagnosed sexual disorders likely reflects underreporting. Findings underscore the need for clinicians to integrate sexuality and relationship safety into ADHD assessment and care.

**Disclosure of Interest:**

None Declared

